# Comparative Neurodynamic Analysis of Spinal Energy Enhancement in Experienced Yoga Practitioners Using Various Breathing Techniques

**DOI:** 10.7759/cureus.73541

**Published:** 2024-11-12

**Authors:** Varun Malhotra, Tanusha Pathak, Danish Javed, Francisco Jose Cidral-Filho, Chanchal Suryawanshi, Mohit Agrawal, Gaurav Mittal

**Affiliations:** 1 Physiology, All India Institute of Medical Sciences, Bhopal, IND; 2 Ayurveda, Yoga and Naturopathy, Unani, Siddha, Sowa-Rigpa, and Homoeopathy (AYUSH), All India Institute of Medical Sciences, Bhopal, IND; 3 Experimental Neuroscience Laboratory (LaNEx), University of South Santa Catarina, Palhoça, BRA; 4 Physical Medicine and Rehabilitation, S.S. Agrawal Institute of Physiotherapy and Medical Care Education, Navsari, IND; 5 Internal Medicine, Mahatma Gandhi Institute of Medical Sciences, Wardha, IND; 6 Research and Development, Student Network Organization, Mumbai, IND

**Keywords:** bioenergetics, breathing exercises, physical fitness, spinal cord, yoga

## Abstract

Introduction: Yoga practices emphasize spinal energy's role in physical, mental, and spiritual well-being, suggesting specific techniques that can enhance energy flow along the spine. Modern research aims to validate these claims and understand the mechanisms behind these effects, potentially integrating them into contemporary healthcare models. This study explores the relationship between yoga breathing techniques, spinal energy dynamics, and health based on yoga philosophy and bioenergetics.

Methodology: Thirty participants, averaging 44 years old with 10-20 years of yoga practice, underwent sessions of slow deep breathing (SDB), alternate nostril breathing (ANB), kapalbhati pranayama, specific nostril breathing exercises, and moderate exercise as a control. Spinal energy was measured using the NeuralChek Spinal Energy system (BrainTap^®^ INC, New Bern, NC), capturing baseline, session, and postsession readings. Statistical analyses assessed changes in spinal energy levels across cervical, thoracic, lumbar, sacral, and coccygeal regions.

Results: SDB and ANB significantly increased spinal energy levels across multiple spinal regions. Kapalbhati pranayama showed variable effects, with significant decreases observed in some regions. Specific nostril breathing exercises also demonstrated significant increases in spinal energy. In contrast, exercise resulted in decreased spinal energy levels, particularly in cervical and lumbar regions.

Conclusion: The findings highlight the potential of yoga breathing techniques to influence spinal energy dynamics, correlating with traditional yoga teachings on energy centers and modern concepts of bioenergetics. These results suggest avenues for integrating yoga practices, particularly breathing techniques, into holistic healthcare approaches aimed at enhancing physiological and psychological well-being. Further research could explore underlying mechanisms and clinical applications, bridging ancient wisdom with contemporary scientific understanding for optimized health outcomes.

## Introduction

The growing interest and research focus on the relationship between yoga practices, breathing techniques, and overall health, underscoring a key concept in yoga philosophy known as "spinal energy" [1]. This notion suggests that the well-being of individuals is intimately tied to the flow of energy along the spine. Bioenergetics, also known as Quantum Medicine, works with human energy fields in various aspects like spiritual, emotional, and psychological realms. It has gained importance with the advancement of science, emphasizing its integration with modern medicine for enhanced therapeutic outcomes [2]. Energy medicine challenges the dominant biochemical paradigm in conventional medicine by offering unique strengths that could enhance traditional healthcare models [3]. Chakras in yoga are often overlooked in scientific research due to their mystical connotations, hindering the understanding of their physiological influence. It is mentioned that intercellular gap junction connections may underlie the subtle energy systems described in yoga, including chakras, offering a scientific basis for these subjective phenomena [4]. The physical aspects of chakras are integrated through gap junction mechanisms, potentially originating during embryological development, and may generate radiant qualities subjectively experienced in chakras [5]. Chakras are suggested to be hubs in dormant electrical circuitry accessible to conscious control, influencing the central nervous system (CNS), autonomic nervous system (ANS), and endocrine system, with practices like yoga providing access to these subtle electrical functions [6]. Various definitions of chakras exist, describing them as psychoenergetic vortices, centers of consciousness, or points of connection between the physical and invisible bodies through which energy flows [7].

“Chakras” are the energy centers located at the midline of the human body, responsible for the energy release of all major physical, mental, and spiritual journeys in human life. The seven primary chakras presented in the human body are Sahasrara (crown chakra), Ajna (third-eye chakra), Vishuddha (throat chakra), Anahata (heart chakra), Manipura (solar plexus chakra), Svadhisthana (sacral chakra), and Muladhara (root chakra) [8]. Subjective experiences described by yoga practitioners can be reproducible through introspective practices, potentially accessible to anyone, highlighting the practicality of understanding chakras' physiological basis [5]. The gap junction theory of chakras offers a mechanism to explain the effects on physical systems claimed by yoga practitioners, emphasizing the importance of considering both physical and metaphysical aspects of chakras [5]. Understanding the physical systems related to chakras, including their influence on glandular secretions and neural control processes, could lead to advancements in technologies like functional magnetic resonance imaging or functional near-infrared spectroscopy to detect signs of this underlying physiology, potentially validating chakra functions [5]. By focusing on gap junction mechanisms and electrical networks over chemical synapses, the paper suggests that advanced meditation practices could restore primitive electrical circuits, enhancing control over the CNS, ANS, and endocrine system, offering a pathway for individuals to access subtle energetic states and broaden their conscious control [6].

The Third Eye (Indigo) and Sacral (Orange) are responsible for connecting to one's spiritual being and enhancing intuition and creativity. They encourage individuals to delve inward to examine emotional responses, ego-self aspects, and emotional beliefs for personal growth by clearing negative thoughts and limiting beliefs to shift perceptions, connect with the heart center, and follow the guidance of the Higher Self [6]. Moreover, each chakra is associated with various physical, emotional, and spiritual aspects of human existence. For instance, Muladhara governs feelings of security and survival, while Svadhisthana influences emotions, sexuality, and creativity. Similarly, Manipura is linked to energy regulation, assimilation, and digestion, and Anahata is associated with love, balance, and wellness. Vishuddha pertains to communication and personal growth, Ajna represents awareness and enlightenment, and Sahasrara correlates with consciousness and liberation. These chakras are commonly understood as centers designated to specific functions across different body regions [9-11]. The objective of the paper is to establish a connection between human physiology's biochemistry and the application of bioenergetic medicine, highlighting the holistic approach to healthcare and well-being [5].

Recent research aims to explore how various yoga exercises and mindful breathing impact this spinal energy and, consequently, one's physiological and mental health, which will help in elucidating the mechanism of how yoga and breathwork contribute to holistic wellness and how practices like yoga postures and controlled breathing techniques influence the body's energy flow and overall health outcomes [5]. Several researchers have linked practices rooted in yoga and meditation to favorable psychological and physiological results among diverse groups, encompassing clinical and nonclinical cohorts. A mounting body of evidence indicates that these practices exert beneficial influences on the regulation of the hypothalamic-pituitary-adrenal axis and inflammatory pathways. Studies have proposed that yoga, alongside other contemplative techniques, could rival pharmacotherapy in effectively addressing mood disorders like anxiety and depression [6].

Holistic energy healing represents traditional alternative therapeutic approaches to reestablish equilibrium and vitality across the body, mind, and spirit. The primary advantage of holistic healing practices lies in their ability to engage with patients on an internal level, facilitating profound healing. Additionally, these modalities address ailments at their root causes, contributing to more comprehensive treatment outcomes. Healers employ a variety of techniques, such as chanting, hands-on healing, prayer, and techniques inducing altered states of consciousness to facilitate healing and restoration [7].

These seven chakras represent focal points within the body, invisible to the naked eye but perceptible through the subtle practice of meditation. Each chakra is believed to correspond to distinct levels of energy, and their alignment and energy flow are pivotal in determining one's overall well-being [4]. Optimal alignment and heightened energy levels within these chakras signify elevated strength and consciousness. Yoga and meditation have captured the interest of contemporary researchers as intriguing subjects of study. Research has highlighted the therapeutic and spiritual advantages inherent in these ancient practices, which are grounded in concepts such as chakras (energy centers), prana (life energy), and nada (sound). Despite their potential insights, there has been a paucity of research in these domains [9-11]. Therefore, the present study seeks to examine the immediate effects of these practices. This research light on the profound effects of such practices on physical and mental well-being, bridging ancient wisdom with contemporary scientific understanding.

## Materials and methods

Study design and setting

The present experimental study was conducted with 30 healthy volunteers at the Department of Physiology at the All India Institute of Medical Sciences (AIIMS), Bhopal, following the necessary ethical approvals from the AIIMS Bhopal Institutional Human Ethics Committee (IHEC-LOP/2021/IM0427). For a medium effect size (Cohen's d = 0.5), with a significance level of 0.05 and 80% power, the required sample size was calculated to be 30 participants.

Selection Criteria

Participants included in the study were required to be regular yoga practitioners for the past 10-20 years, ensuring substantial experience with yoga. Those with any known health conditions that could affect the study outcomes were excluded. This approach aimed to maintain a homogeneous group and ensure the reliability and validity of the results.

Data sources and variables

Participants were given at least 10 minutes to rest before data collection, and they wore comfortable clothing. The room temperature was maintained in a thermoneutral zone, and recordings were conducted in silence. Spinal energy was analyzed using the NeuralChek Spinal Energy system (BrainTap® INC, New Bern, NC; Dinamika, Dinamica S.r.l., Messina, Italy), which is a novel digital analyzer that measures spinal energy through neurodynamic analysis. The Dinamika/Neuralchek software and hardware adhere to the standards for measurement, physiological interpretation, and clinical application of cardiac intervalometer indices, as established by the European Society of Cardiology and the North American Society of Electrophysiology [12,13]. The system provides real-time monitoring of functional state indices and analyzes the human heart rate extracted from an electrocardiogram (ECG) recording. Physiological parameters can be assessed within five minutes using this system. Two electrodes were placed on the wrist with water or gel, and baseline recordings were taken for five minutes. Subsequent recordings were taken when the yogi practitioner was performing the meditation session. ECG recordings were taken using the NeuralChek system.

Slow Deep Breathing

During the recordings, participants were seated on a chair or cross-legged on a cushion or mat, with a straight spine, shoulders back, abdomen comfortably drawn in, and chin parallel to the ground. Their gaze was directed toward the point between their eyebrows. The recording session lasted for 10 minutes, consisting of five minutes of resting baseline and five minutes during the slow deep breathing (SDB) maneuver. The procedure for SDB involved slow and deep inhalation through the nose, typically to a count of 15, followed by breath holding for an equal count of 15, and then slow and complete exhalation for the same count. This process was repeated throughout the five-minute breathing maneuver. If participants found it challenging to hold the count to 15, they were instructed to reduce the counting to a comfortable level during each phase of the exercise. Counting in this breathing exercise was paced at approximately two counts per second, resulting in a breathing rate of two to four breaths per minute.

Alternate Nostril Breathing

Participants were asked to maintain a specific posture, sitting on a chair or cross-legged on a cushion or mat, with a straight spine, shoulders back, abdomen comfortably drawn in, and chin parallel to the ground. Their gaze was directed toward the point between their eyebrows. The recording session lasted 15 minutes, comprising five minutes of resting baseline and five minutes during the alternate nostril breathing (ANB) maneuver. The procedure for the ANB involved several steps: participants closed one nostril (e.g., the right nostril) with their thumb and slowly inhaled through the other nostril (left nostril) for a maximum count of 25. Then, they held their breath for a comfortable count of 6 or 12. Next, they closed the opposite nostril (left) with their ring finger, opened the initial nostril (right), and exhaled slowly through it for a maximum count of 25. The process was then reversed, with inhalation through the same nostril (right) and exhalation through the other (left). Counting during the breathing exercise was paced at approximately two counts per second.

Kapalbhati Pranayama

The kapalbhati pranayama was conducted under the supervision of a qualified yoga practitioner. Participants were seated in a comfortable position with a straight spine, relaxed body, and shoulders drawn back. They were instructed to sit on a chair with a straight spine, draw in their stomach, bring their shoulder blades together, and keep their chin parallel to the ground. Kapalbhati pranayama involved deep inhalation followed by forceful exhalation with rapid movements of the diaphragm and abdominal muscles. This procedure was performed for five minutes. Due to its vigorous nature and potential risks, including pneumothorax reported in a case study, caution was exercised, and the duration was limited to five minutes. It was emphasized that this practice should only be done under expert supervision and consultation.

Right and Left Nostril Breathing

Before data collection, volunteers were given a minimum of 10 minutes to rest and were dressed comfortably. The ambient room temperature was maintained within the thermoneutral range, and silence was observed during data acquisition. Pranayama exercises were performed before meals, with participants instructed not to hold their breath for extended periods to avoid undue pressure on vital organs. Individuals with lung conditions such as pulmonary tuberculosis, interstitial lung disease, lung fibrosis, or vertebral deformities like kyphosis and scoliosis that might hinder comfortable breath-holding were excluded from participation.

Surya Nadi and Chandra Nadi Pranayama

Surya nadi pranayama began by closing the right nostril using the thumb of the left hand, exhaling through the right nostril, and inhaling slowly through the same nostril, constituting one round. Inhalation lasted for a count of 25, holding the breath for a count of 12, and exhalation for a count of 25. The counting rate was set at two counts per second, making one round approximately 30 seconds long. Heart rate variability was recorded before and during each pranayama session, with inhalation and exhalation exclusively through the right nostril referred to as surya bhedana pranayama. An alternative variation called surya anuloma viloma pranayama exclusively involved inhalation and exhalation through the right nostril. In contrast, chandra bhedana pranayama involved inhalation through the left nostril and exhalation through the right nostril exclusively. A variation known as chandra anuloma viloma pranayama exclusively entailed inhalation and exhalation through the left nostril. Chandra nadi pranayama commenced with closing the right nostril using the thumb of the right hand, followed by exhaling through the left nostril and inhaling slowly through the same nostril, forming one round of chandra nadi suddhi pranayama. The inhalation, holding, and exhalation counts were identical to those in surya nadi pranayama.

Exercise Protocol

Initially, study participants were given a five-minute rest period during which spinal energy was recorded. Subsequently, they underwent warm-up and stretching exercises for 5-10 minutes. Following the warm-up, participants engaged in stationary cycling exercise at a mild intensity (30%-50% of maximal heart rate) for five minutes. Exercise was halted if the participant experienced discomfort or if there was a sudden increase in heart rate. Spinal energy recording was performed during the preexercise resting period, at five-minute intervals during the 20-minute exercise, and during the postexercise recovery period.

Statistical analysis

Data were entered into a master graphic using Microsoft Excel (Microsoft Corporation, Redmond, WA) and analyzed with SPSS version 20 (IBM Corp. Armonk, NY). Descriptive statistics, including frequency analysis, percentage analysis, and mean analysis, were used to characterize categorical and continuous variables. Statistical information was presented as mean ± standard deviation.

## Results

Table 1 shows the effectiveness of different breathing techniques in enhancing spinal energies at various levels. The studies comparing mean values of different plexus points before and during intervention showed significant changes across five chakras. For the cervical plexus, significant increases were noted in studies 1, 2, 4, and 5 (p < 0.0001), with effect sizes ranging from 1.56 to 4.77, except for study 3, which showed a decrease with a p value of 0.0514. In the thoracic plexus, all studies except study 6 showed significant increases (p < 0.0001), with effect sizes between 0.31 and 2.50. Lumbar plexus studies consistently showed significant increases (p < 0.0001), with effect sizes ranging from 1.02 to 5.55. The sacral plexus showed significant increases in all studies (p < 0.0001) except study 6 (p = 0.0266), with effect sizes from 0.75 to 3.47. For the coccygeal plexus, significant increases were observed in all studies (p < 0.0001), with effect sizes ranging from 0.58 to 4.84.

**Table 1:** The effectiveness of different breathing techniques to enhance the spinal energies at various levels Effect sizes (95% confidence interval); study 1: slow deep Kriya breathing (n = 24), study 2: alternate nostril breathing, study 3: kapalbhati (fast breathing), study 4: right nostril breathing, study 5: left nostril breathing, study 6: exercise (n = 20; studies 2-6) A p value of <0.05 is considered to be statistically significant SD: standard deviation; SE: standard error

Chakras/plexus	Mean ± SD (before)	Mean ± SD (during)	p value	Effect size r	SE
Cervical					
Study 1	42.42 ± 16.12	97.75 ± 3	<0.0001	4.772183	3.347
Study 2	33.10 ± 15.5	77.10 ± 14.99	<0.0001	2.885789	4.822
Study 3	9.15 ± 5.93	5.80 ± 3.71	0.0514	0.677293	1.564
Study 4	52.17 ± 15.15	96.11 ± 6.61	<0.0001	3.759439	3.696
Study 5	46.65 ± 18.03	94.10 ± 8.85	<0.0001	3.341038	4.491
Study 6	48.35 ± 12.83	26.75 ± 14.73	<0.0001	1.563777	4.368
Thoracic					
Study 1	62.00 ± 20.55	98.67 ± 2.33	<0.0001	2.507497	4.222
Study 2	45.40 ± 21.49	81.85 ± 18.75	<0.0001	1.807445	6.377
Study 3	42.95 ± 22.65	30.85 ± 3.25	0.0070	0.652111	5.868
Study 4	67.67 ± 22.11	97.50 ± 5.68	<0.0001	1.847999	5.104
Study 5	62.80 ± 27.85	95.05 ± 7.78	<0.0001	1.577257	6.466
Study 6	56.25 ± 19.25	41.90 ± 61.94	0.3673	0.312877	14.504
Lumbar					
Study 1	55.71 ± 10.46	98.29 ± 2.88	<0.0001	5.550363	2.215
Study 2	47.95 ± 11.53	79.45 ± 13.33	<0.0001	2.527575	3.941
Study 3	57.50 ± 29.22	33.10 ± 16.64	0.0002	1.026199	7.519
Study 4	61.83 ± 10.74	96.94 ± 4.83	<0.0001	4.216425	2.633
Study 5	53.45 ± 14.02	97.55 ± 4.24	<0.0001	4.257959	3.275
Study 6	54.35 ± 8.82	30.75 ± 16.59	<0.0001	1.776344	4.201
Sacral					
Study 1	44.63 ± 19.35	94.63 ± 6.45	<0.0001	3.466772	4.163
Study 2	34.90 ± 17.19	67.90 ± 18.34	<0.0001	1.856613	5.621
Study 3	42.55 ± 23.62	25.60 ± 13.68	0.0024	0.878199	6.103
Study 4	52.44 ± 21.13	88.50 ± 10.48	<0.0001	2.162138	5.274
Study 5	44.45 ± 20.27	86.95 ± 11.55	<0.0001	2.576289	5.217
Study 6	46.90 ± 15.79	36.30 ± 12.29	0.0266	0.749188	4.474
Coccygeal					
Study 1	52.43 ± 13.38	98.96 ± 2.44	<0.0001	4.838247	2.776
Study 2	44.15 ± 14.94	75.20 ± 14.85	<0.0001	2.084583	4.710
Study 3	49.80 ± 18.53	23.25 ± 11.65	<0.0001	1.715434	4.894
Study 4	62.56 ± 12.00	97.06 ± 5.18	<0.0001	3.732922	2.923
Study 5	51.70 ± 17.86	97.45 ± 4.45	<0.0001	3.515166	4.116
Study 6	27.60 ± 17.01	37.20 ± 15.97	0.0020	0.581882	5.217

Table 2 describes the omnibus test of model coefficients and test of residual heterogeneity for different spinal levels using fixed-effects methods. For the cervical plexus, the omnibus test showed Q = 2.544 (p = 0.111) and residual heterogeneity with Q = 1.751 (p = 0.882). In the thoracic plexus, the omnibus test had Q = 0.558 (p = 0.455) and residual heterogeneity with Q = 0.078 (p = 1.000). The lumbar plexus showed a significant omnibus test with Q = 10.080 (p = 0.001) and residual heterogeneity with Q = 1.063 (p = 0.957). For the sacral plexus, the omnibus test had Q = 1.014 (p = 0.314) and residual heterogeneity with Q = 0.249 (p = 0.998). Finally, in the coccygeal plexus, the omnibus test showed Q = 4.959 (p = 0.026) and residual heterogeneity with Q = 0.770 (p = 0.979). Note that p values are approximate.

**Table 2:** Omnibus test of model coefficients and test of residual heterogeneity p values are approximate. The model was estimated using the fixed-effects method A p value of <0.05 is considered to be statistically significant df: degrees of freedom; Q: test statistic

Fixed and random effects	df	Cervical		Thoracic		Lumbar		Sacral		Coccygeal	
		Q	p	Q	p	Q	p	Q	p	Q	p
Omnibus test of model coefficients	1	2.544	0.111	0.558	0.455	10.080	0.001	1.014	0.314	4.959	0.026
Test of residual heterogeneity	5	1.751	0.882	0.078	1.000	1.063	0.957	0.249	0.998	0.770	0.979

## Discussion

The human body is a complex interplay of physiological systems, and recent research delves into the fascinating connection between yogic practices and energy levels within the spine. This study investigates the concept of "spinal energy," measured using a device called NeuralChek SPINAL ENERGY, and explores how various breathing exercises and yoga techniques influence these energy levels across different spinal regions. The study design incorporates multiple experiments, each focusing on a specific breathing or yoga practice. Participants perform slow deep Kriya breathing, ANB (Ananda Brahman), kapalbhati (fast breathing), right nostril breathing (Surya Bhedana), left nostril breathing (Chandra Bhedana), and even standard physical exercise. Before and during each practice, spinal energy is measured in the cervical, thoracic, lumbar, sacral, and coccygeal regions.

The findings reveal intriguing insights. Slow, deep kriya breathing and ANB lead to significant increases in spinal energy across most measured regions. This suggests a potential for these practices to enhance energy flow and physiological well-being [6]. Interestingly, kapalbhati has the opposite effect, causing a decrease in spinal energy in the thoracic, lumbar, sacral, and coccygeal regions, with the cervical region showing no significant change. This particular finding warrants further investigation into the targeted effects of kapalbhati on specific areas of the spine [7]. Further studies explore the impact of right and left nostril breathing, revealing similar increases in spinal energy across all measured regions for both practices. This suggests that both techniques might be beneficial for promoting energy balance within the spine [8]. Finally, the study examines the effects of exercise on spinal energy. Here, the results show a decrease in energy levels in the cervical, lumbar, and coccygeal regions, while the thoracic region exhibits no significant change. This differential response across the spine during exercise highlights the need for further exploration into the intricate relationship between physical activity and energy distribution within the body [9]. Overall, this research paves the way for a deeper understanding of how yogic practices and breathing exercises can influence what is termed "spinal energy." The observed changes in energy levels across different spinal regions point toward potential benefits for overall well-being and physiological functioning. Future studies can delve deeper into the mechanisms underlying these changes, exploring the immediate effects and long-term implications of these practices on human health and performance.

Kriya yoga is a spiritual practice designed to accelerate spiritual growth and deepen meditation through techniques that increase and harmonize energy flow within the body, with a key focus on spinal energies [10]. In yogic traditions, the spine is considered the central channel for energy flow, known as the Sushumna Nadi, flanked by the Ida and Pingala Nadis. The core technique, Kriya Pranayama, involves specific breathing exercises that move energy up and down the spine, synchronized with mental focus on the chakras (energy centers) to purify energy channels and enhance life force flow [11]. Emphasizing the chakras along the spine, practitioners concentrate on activating and balancing Muladhara (base), Swadhisthana (sacral), Manipura (navel), Anahata (heart), Vishuddha (throat), Ajna (third eye), and Sahasrara (crown) chakras during meditation and breathing exercises. Meditation techniques often include visualizations of energy rising up the spine from the base to the crown chakra, aided by mantras and affirmations to deepen the meditative state and awareness of energy flow. Bandhas (body locks) and mudras (hand gestures), such as Mula Bandha (root lock), Uddiyana Bandha (abdominal lock), and Jalandhara Bandha (throat lock), are employed to control and direct prana flow, establishing a continuous circulation of energy up and down the spine. This circulation is believed to cleanse and activate the chakras, leading to a balanced and heightened state of awareness [14]. The enhanced Spinal energy flow is thought to improve overall vitality, mental clarity, and emotional stability, contributing to physical and emotional healing by harmonizing the energy body. Ultimately, Kriya yoga aims for spiritual awakening, realizing one's true nature or union with the divine through a purified spine, which facilitates deeper meditation and profound spiritual experiences. Practitioners can potentially unlock deeper levels of consciousness and experience significant spiritual transformations by understanding and applying these techniques [15].

Kapalbhati Pranayama, a vigorous breathing technique characterized by short, forceful exhalations and passive inhalations, can temporarily decrease spinal energies due to specific physiological mechanisms [16,17]. The rapid, forceful exhalations lower carbon dioxide levels in the blood, causing vasoconstriction and reducing blood flow to the brain and spinal cord, thereby affecting the oxygen supply and energy levels along the spine. Additionally, the sympathetic nervous system may become temporarily activated due to the intense nature of the breathing, leading to heightened alertness followed by a brief decrease in energy as the body readjusts [18]. The cleansing nature of kapalbhati, which focuses on expelling toxins and stale air from the lungs, can also result in energy being redirected away from the spinal pathway to support detoxification efforts. This redistribution can lead to a short-term decrease in the energy available to flow through the spine [19]. However, once the practice is complete, the body and energy systems enter a rebalancing phase, normalizing prana levels and blood chemistry, often resulting in increased clarity, lightness, and vitality as spinal energies are restored [20].

ANB, or nadi shodhana, is a yogic practice that involves breathing through one nostril at a time, either left, right, or alternating. This technique is believed to balance the flow of prana (life force) through the body, specifically affecting the Ida and Pingala Nadis, which correspond to the left and right nostrils [21,22]. Breathing through the left nostril (Ida Nadi activation) promotes relaxation and a state of calmness by stimulating the parasympathetic nervous system, reducing stress, and creating a conducive environment for energy to flow smoothly along the spine. Conversely, breathing through the right nostril (Pingala Nadi activation) increases alertness, focus, and energy levels by activating the sympathetic nervous system, enhancing the flow of prana and energizing the body. Alternating between nostrils (nadi shodhana) harmonizes these effects, balancing the sympathetic and parasympathetic nervous systems and optimizing the overall energy flow through the body and along the spinal pathway [8].

This practice also purifies the nadis (energy channels), particularly the Sushumna Nadi, by clearing blockages and balancing the flow of prana through the Ida and Pingala Nadis. Improved oxygen intake and enhanced blood circulation from controlled, deep breathing ensure that the spinal cord and associated energy centers receive an adequate supply of nutrients and oxygen essential for maintaining and increasing spinal energies. Additionally, focusing on the breath and energy flow helps activate and balance the chakras along the spine, while the meditative state induced by ANB enhances mental focus and awareness, effectively channeling prana along the spine and contributing to spiritual growth and well-being [23].

Limitations of the study

One primary limitation of this study is its complexity due to the intricate interplay of physiological systems and the challenge of accurately measuring "spinal energy" using the NeuralChek SPINAL ENERGY device, which may introduce variability. The study design, involving multiple breathing exercises and yoga techniques, complicates the isolation of each practice's effects. Additionally, the small sample size and observational nature limit the generalizability and reliability of the findings, while the absence of a control group hampers the ability to compare outcomes.

## Conclusions

In conclusion, the present study sheds light on the intricate relationship between yogic practices, breathing techniques, and spinal energy levels. It was observed that slow deep kriya breathing and ANB generally increased Spinal energy, while kapalbhati led to decreases in certain regions. Right and left nostril breathing exhibited similar effects, raising energy levels across the spine. Exercise, on the other hand, resulted in decreased energy levels in some regions. These findings underscore the potential benefits of targeted yoga and breathing practices for holistic well-being and physiological functioning. Further research is needed to delve into the underlying mechanisms and long-term implications of these observed effects on human health and performance.
